# HER2-Positive Early Breast Cancer: Time for Ultimate De-Escalation?

**DOI:** 10.3390/cancers16061121

**Published:** 2024-03-11

**Authors:** Nikolas Tauber, Christoph Cirkel, Anna Claussen, Franziska Fick, Emmanuel Kontomanolis, Natalia Krawczyk, Achim Rody, Maggie Banys-Paluchowski

**Affiliations:** 1 Department of Gynecology and Obstetrics, University Hospital Schleswig-Holstein, Campus Lübeck, 23538 Lübeck, Germany; 2 Department of Gynecology and Obstetrics, Democritus University of Thrace, 68100 Alexandroupolis, Greece; 3 Department of Gynecology and Obstetrics, University Hospital Düsseldorf, 40225 Düsseldorf, Germany

**Keywords:** HER2-positive, breast cancer, systemic de-escalation, chemo-free therapy, targeted therapy

## Abstract

**Simple Summary:**

In the quest for de-escalation strategies, the complete elimination of non-targeted cytotoxic chemotherapy is often considered the ultimate goal in oncological therapy. This is particularly evident in HER2-positive breast cancer, where expanding targeted anti-HER2 therapy is a focal point of the current research due to the groundbreaking success achieved with trastuzumab. At present, several ongoing studies are examining chemotherapy-free regimens as part of initiatives to minimize the morbidity and toxic side effects of HER2-positive early breast cancer, while ensuring high oncogenic safety. Despite the promising early results achieved by these studies, the standard of care still entails combining anti-HER2 treatment with a chemotherapy backbone.

**Abstract:**

De-escalation is currently taking place in both the surgical and systemic treatment of breast cancer. The introduction of trastuzumab, the first monoclonal antibody against the HER2 receptor, over 20 years ago was a milestone in the treatment of HER2-positive breast cancer and marked the beginning of a new era in targeted tumor therapy. In the sense of de-escalation, omitting non-targeted cytotoxic chemotherapy altogether is often hailed as the ultimate goal of oncological research. Especially in cases of small, node-negative, HER2-positive early breast cancer, it remains a challenge for clinicians to establish the safest and most efficient treatment plan while considering the significant potential for toxic side effects associated with chemotherapy and HER2-targeted therapy, and the generally excellent prognosis. In this context, several ongoing studies are currently assessing chemotherapy-free regimens as part of strategies aimed at de-escalating therapy in the field of HER2-positive early breast cancer. Despite the promising early results of these studies, the combination of anti-HER2 treatment with a chemotherapy backbone remains the standard of care.

## 1. Introduction

Breast cancer (BC) is the most common cancer in women worldwide, and every eighth woman in the western world is affected by it during her lifetime [1]. The identification of the nuclear estrogen receptor (ER) and the differentiation between ER-positive and ER-negative breast carcinomas in the 1970s by the American scientist Elwood Jensen marked a milestone and the beginning of a tumor biological approach in oncology, from which the still-existing anti-estrogen therapy originated. This development continued in the 1980s and 1990s when another important cellular factor, the human epidermal growth factor receptor (HER), was first described by Shih et al. [2], leading to the development of targeted antibody-based therapy, primarily initiated by the German biochemist Axel Ullrich and the U.S. American oncologist Dennis Slamon [3].

The HER2 receptor is a 1255 amino acid glycoprotein belonging to a family of transmembrane tyrosine kinases, with its coding proto-oncogene located on the long arm of chromosome 17 (q21) [4]. Ligand binding is only possible after the receptor forms heterodimers with other HER receptors and is regulated by autophosphorylation of the HER2 tyrosine kinase, subsequently regulating the Ras-MAPK pathway [5]. HER2 overexpression, due to an increased rate of heterodimer formation and reduced endocytosis, leads to upregulation of the downstream Ras-MAPK pathway, resulting in enhanced cell growth and the inhibition of pro-apoptotic factors in the tumor cell, making it more biologically aggressive. HER2 gene amplification, first described in BC cells in 1985 [6], results in an up to 45-fold increase in receptor density on the cell membrane [7,8]. Since then, activating point mutations, which intensify the signaling cascade without the presence of gene amplification and receptor overexpression, have also been described [9].

In 1987, Slamon et al. reported that the amplification of the Her-2/neu oncogene is correlated with increased relapse risk and reduced survival after BC in humans [10]. They stated that Her-2/neu amplification had a greater prognostic value than other prognostic factors used at that time, such as hormone receptor status and lymph node disease [10]. An aggressive disease biology, such as enhanced cell proliferation and reduced progression-free survival (PFS) and overall survival (OS), was associated with the amplification of the HER2 gene [11,12,13]. Back then, types of BC considered “aggressive” were generally treated with chemotherapy. However, this strategy did not lead to marked improvements in patient survival, as median PFS after the diagnosis of distant disease was as low as 4.6 months and OS was 20 months in patients treated with chemotherapy alone [14]. In this context, identification of the HER2 receptor as a potential target for therapy was a gamechanger.

The introduction of trastuzumab, the first monoclonal antibody against the HER2 receptor, over 20 years ago was a milestone in the treatment of HER2-positive breast carcinomas [15]. Trastuzumab binds to the extracellular domain of the HER2 receptor [16]. The prevention of receptor dimerization, immune activation, increased endocytotic receptor destruction, as well as inhibition of the shedding of the extra-cellular domain, are possible mechanisms of trastuzumab receptor binding [17]. Its introduction marked the beginning of a new era in targeted tumor therapy and was followed by the approval of monoclonal antibodies in nearly all oncological entities. The name trastuzumab is derived from the last three syllables, “-tu” “-zu” and “-mab”, which, according to the established nomenclature for monoclonal antibodies, indicate both therapeutic use against tumors (“-tu”) and the biochemical structure as a humanized (“-zu”) monoclonal antibody (“-mab”) [18].

While there have been further developments in anti-HER2 therapy, such as the introduction of a dual blockade with trastuzumab/pertuzumab or trastuzumab emtansin (T-DM1) as the first antibody–drug conjugate (ADC) for BC, all of these developments have followed the principle of therapy targeting HER2 overexpression, as the use of trastuzumab in tumors other than those that were HER2-positive did not show a clinical benefit.

HER2-enriched tumor cells are defined by a strong immunohistochemical (IHC) staining reaction (3+). In the case of moderate staining (2+), an additional positive confirmation using in situ hybridization (ISH) is required. According to the current pathological classification, all 2+ tumors with negative ISH or low to absent immunohistochemical staining (1+/0) are considered HER2-negative [19,20]. However, a significant number of tumors exhibit low to moderate expression of HER2 (2+ with negative ISH/1+), which have traditionally been treated according to the recommendations for HR+ HER2-negative or triple-negative breast cancer (TNBC) [21]. Recently, the DESTINY-Breast 04 trial has shown a significant clinical benefit of therapy with ADC trastuzumab deruxtecan (T-DXd) in patients with advanced breast cancer (aBC) with low HER2 expression. As a result, T-DXd is currently approved for use in both HER2-low and HER2-enriched tumors [22]. It is worth noting that, due to the existing limitations and uncertainties in the exact definition of the term HER2low, the ASCO–College of American Pathologists (CAP) does not recommend this term for the mentioned tumors. Instead, it is recommended to include a reporting footnote for tumors that may be eligible for a treatment targeting non-amplified/non-overexpressed levels of HER2 expression for cytotoxic drug delivery [19].

## 2. HER2-Positive Early Breast Cancer: “From no Hope to Excellent Prognosis in 20 Years”

For decades, HER2-overexpressing early breast cancer (eBC) was associated with poor outcomes and higher mortality rates compared with other BC subtypes. However, following the discovery of the HER2 receptor as a potential target for personalized therapy, the development of a specific antibody immediately improved the therapy options and prognosis of that subtype of BC. The benefit of trastuzumab in the setting of eBC was shown in several large, randomized trials [23,24,25] and a new standard in the treatment of HER2-positive BC was set. FDA approval for trastuzumab was granted in the U.S. in 1998, making trastuzumab the first approved antibody for the treatment of HER2-positive BC (Table 1).

The second monoclonal antibody that was discovered and used was pertuzumab, which binds at a different epitope of the extracellular domain [32]. Pertuzumab prevents HER2 from dimerization with other receptors from the EGFR/ErbB group, especially the HER3 receptor [33,34]. The most potent signaling heterodimer is considered to be the HER2/HER3 heterodimer, which strongly promotes cell proliferation in HER2-positive cancer [34]. 

The NeoSphere phase II study showed that pertuzumab and trastuzumab, administered together in combination with neoadjuvant chemotherapy, significantly increased the rate of pathological complete response (pCR) in HER2-positive BC patients [28]. Because of the binding to different HER2 epitopes, trastuzumab and pertuzumab have a complementary mechanism of action, providing a more comprehensive blockade of the HER2 signaling pathway and a greater antitumor activity than each antibody administered alone [35]. The APHINITY trial was a phase III study that demonstrated that adjuvant pertuzumab, in combination with trastuzumab and chemotherapy, significantly improved invasive disease-free survival (iDFS) among patients with operable HER2-positive BC [27,36]. 

While the prognosis of BC has significantly improved since the introduction of HER2-targeted therapy, the risk of disease recurrence and death remains high in patients with residual invasive disease at surgery, especially when compared to those who achieved pCR through neoadjuvant chemotherapy [28,37,38,39,40]. The ADC T-DM1 consists of trastuzumab and the microtubule inhibitor emtansine as a cytotoxic agent [41]. After binding to the HER2 receptor, trastuzumab emtansine is internalized into the BC cell and undergoes proteolytic degradation, releasing the active cytotoxic agent emtansine and resulting in cell death [41]. The efficacy of T-DM1 in the post-neoadjuvant setting was examined in the KATHERINE trial [29]. A total of 1486 patients with HER2-positive BC and residual invasive disease in the breast or axilla after standard neoadjuvant treatment were randomly assigned to receive 14 cycles of either T-DM1 or trastuzumab. The final iDFS and updated OS analysis were recently presented at the San Antonio Breast Cancer Symposium (SABCS) 2023 [30]. This analysis revealed a significantly improved OS after 7 years (89.1% in the T-DM1 arm vs. 84.4% in trastuzumab arm, hazard ratio (HR) 0.66; 95% CI 0.51, 0.87; *p* = 0.0027). The final iDFS benefit after 7 years of T-DM1 was sustained in the intention-to-treat population (80.8% vs. 67.1%, HR 0.54, 95% CI 0.44, 0.66). While T-DM1 was associated with a higher incidence of adverse events (98.8% vs. 93.3%) and a higher rate of treatment discontinuation (18% vs. 2.1%) compared to trastuzumab, it is worth noting that the regimen is generally well tolerated and has a lower toxicity than conventional monochemotherapies. Based on the KATHERINE trial, T-DM1 was the first evidence-based post-neoadjuvant strategy to show an improved clinical outcome in BC patients, leading to an increase in the use of neoadjuvant therapy [30]. 

A different way to interact with the HER2 pathway is via acting on intracellular tyrosine kinase domains. Neratinib, as a small molecule, inhibits EGFR, HER2 and HER4 tyrosine kinase. The reduction in EGFR and HER2 autophosphorylation and downstream signaling results in a reduction in the growth of EGFR- and HER2-dependent cell lines. The binding of neratinib to the targeted kinase is irreversible [42]. Compared to trastuzumab and pertuzumab, where extracellular binding to the receptor occurs, neratinib exhibits intracellular effects in BC cells, which became resistant to trastuzumab treatment [43] or are co-activated through EGFR signaling [44]. The ExteNET trial showed that neratinib administered for 1 year improved iDFS in the HR-positive HER2-positive (sometimes referred to as “triple positive”) patient population after trastuzumab treatment [31].

Currently, the research focus is on molecular markers that can predict treatment response before the initiation of therapy. Some recently described examples include immune signatures (adaptive immune signatures) that allow for the estimation of the likelihood of achieving a pCR solely with anti-HER2 therapy. Patients with a low expression of the immune signature, on the other hand, more frequently exhibited a non-pCR [45]. In the future, it could be possible to identify patients who would benefit from an escalated therapeutic strategy, such as dual anti-HER2 blockade, only through molecular tumor markers.

## 3. Post-Neoadjuvant Therapy: Time for New Players?

NACT allows for the evaluation of tumor responsiveness to treatment in vivo, enabling adjustments to post-neoadjuvant therapy based on the pathological response to NACT. The achievement of a pCR is particularly significant in HER2-positive breast cancer, as it is associated with enhanced long-term outcomes chemotherapy [28,37,38,39,40].

In further investigations involving molecular characterization from the data of the NeoALTTO study [39,46] it is observed that immune metagenes and the composition of tumor-infiltrating tumor cells within the tumor microenvironment vary depending on the treatment response. These variations serve as predictive markers for treatment responsiveness. The molecular identification of responsive patients during NACT becomes feasible, making the integration of immune profiling for the characterization of the primary tumor a promising approach [47].

One of the goals of modern cancer research is to find strategies that will effectively combat tumor cells with as little toxicity as possible to other rapidly dividing cells. Therefore, numerous trials over the past few years have focused on drugs designed to increase the transfer of cytotoxic compounds to cancer cells by using antibodies targeting the antigens presented on those cells. In this context, ADCs are still a relatively new group of cancer-targeted drugs, of which only a small fraction have been tested in large clinical trials at present.

T-DM1 was the first representative of this group to be approved for the treatment of a solid tumor. As an ADC, it consists of a cytotoxic agent (emtansine) and an antibody (trastuzumab) connected through a stable thioether linker. Earlier studies have shown that all three of the components (the antibody, linker, and cytotoxic payload) that make up the structure of ADCs have a critical impact on therapy outcomes. Changing even just the linker itself results in significant differences in the pharmacokinetics, anticancer activity, and toxicity of the drug [41].

Besides the T-DM1, there are two other ADCs currently approved for BC therapy in Europe. Trastuzumab deruxtecan (T-DXd) is an antibody against HER2 (trastuzumab), conjugated with a topoisomerase I inhibitor (deruxtecan, a derivative of exatecan) through tetrapeptide-based linker, approved for the treatment of HER2-positive as well as HER2-low metastatic BC. Sacituzumab govitecan, an antibody against Trop-2, conjugated with the active metabolite of irinotecan, SN-38, is currently approved for the treatment of metastatic triple-negative and HR-positive HER2-negative BC [48].

While T-DM1 remains the only approved post-neoadjuvant strategy for HER2-positive patients with non-pCR following neoadjuvant therapy, studies on the efficacy of new drugs in this setting are currently underway. The multicenter open-label randomized TruDy/DESTINY-Breast05 study aims to compare the efficacy of T-DXd with T-DM1 as a post-neoadjuvant treatment in HER2-positive patients with invasive tumor rest [49]. Similar to the KATHERINE study, the patients included in this trial must have received at least 16 weeks of taxane and trastuzumab before surgery. The recruitment is scheduled to close in 2024. The rationale for this study was the superior outcome of T-DXd, compared to T-DM1, achieved in the metastatic setting. In the DESTINY-Breast03 study, patients with HER2-positive metastatic BC were randomized to T-DXd vs. T-DM1 [50]. The trial showed significantly longer OS and PFS in the T-DXd arm (OS: HR 0,64; *p* = 0.0037; 2-year OS 77.4% vs. 69.9%; median PFS: 28.8 vs. 6.8 months, HR 0.33; *p* < 0.0001). 

While maintaining an optimistic approach to trials on further applications of the antibody–drug conjugates, it is important to keep in mind the potentially increased toxicity of these drugs, which goes hand in hand with enhanced antitumor activity. When using T-DXd, pulmonal toxicity is an adverse event of special interest and treatment-related deaths have been reported in earlier studies. In the DESTINY-Breast03 trial, more patients discontinued treatment in the T-DXd group (20% vs. 7% in the T-DM1 group), and more dose reductions (25 vs. 15%), as well as drug interruptions due to adverse events (42% vs. 17%), were observed in the T-DXd arm. The most frequent reasons for the discontinuation were pneumonitis, interstitial lung disease, and pneumonia. Drug-related interstitial lung disease or pneumonitis occurred in 15% of patients treated with T-DXd and only 3% of patients treated with T-DM1 [50]. However, no therapy-related deaths were reported, suggesting an improved toxicity management and the importance of a learning curve compared to previous studies.

## 4. Treatment De-Escalation: Who Is “Low Risk”? 

De-escalation is currently taking place in both the surgical and systemic treatment of BC. In both the surgical and systemic therapy settings, it is essential to strike the right balance between benefits and side effects. The aim of this is to improve the quality of life while maintaining a high level of oncological safety and, at the same time, reducing treatment-related morbidity. The introduction of sentinel lymph node biopsy is considered to be a milestone in de-escalating surgical interventions for BC patients. Currently, there are varying guideline recommendations regarding the omission of sentinel lymph node biopsy (SLNB) in older patients with low-risk tumors [51,52]. Additionally, one of the most controversially discussed settings in surgical treatment remains the optimal approach for patients who initially had a positive node status but achieved a complete axillary response (cN+ → ycN0) after neoadjuvant chemotherapy [53,54,55]. 

The issue of axillary staging in clinically node-negative (cN0) patients undergoing NACT is still awaiting clarification. The ongoing, prospective, single-arm EUBREAST-01 study (*n =* 334) is currently investigating the oncological safety of omitting SLNB in patients who are clinically node-negative at the time of diagnosis and achieve a pCR of the breast (ypT0) during NACT. Patients enrolled in this study have triple-negative or HER2-positive breast carcinomas, as previous research suggests that breast pCR is indicative of axillary pCR in these subtypes [56,57].

The right balance between appropriate systemic therapy and avoiding unnecessary side effects to enhance the quality of life is particularly important in the case of low-risk tumors. Especially in case of small, node-negative, HER2-positive eBC, it remains a challenge for clinicians to establish the safest and most efficient treatment plan while considering the significant potential for the toxic side effects associated with chemotherapy and HER2-targeted therapy, and the generally excellent prognosis. While several trials demonstrated improved clinical outcomes in patients treated with trastuzumab and multiagent chemotherapy, it is worth noting that the number of patients with small node-negative tumors was very low [23,25,27,58].

**Figure 1:**
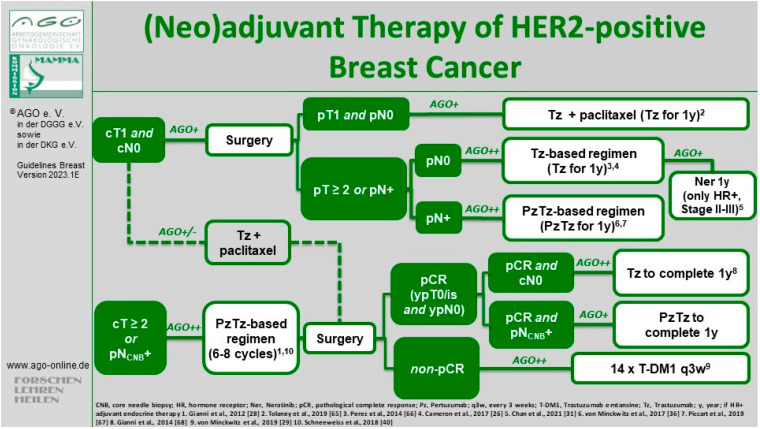
Current treatment algorithm for HER2-positive eBC by the AGO Breast Committee [64]. © AGO e. V. Guidelines Breast Version 2023.1E. https://www.ago-online.de/leitlinien-empfehlungen/leitlinien-empfehlungen/kommission-mamma (latest update 15 March 2023). Reproduced with permission from AGO Breast Committee. “1” represents [28], “2” represents [65], “3” represents [66], “4” represents [26], “5” represents [31], “6” represents [36], “7” represents [67], “8” represents [68], “9” represents [29], “10” represents [40].

The open-label single-arm, multicenter, phase II Adjuvant Paclitaxel and Trastuzumab (APT) trial aimed to address this issue [59]. The study enrolled 406 patients with small (≤3 cm) HER2-positive eBC between 2007 and 2010. All patients received primary surgery and were either node-negative or had micrometastatic nodal involvement (N1mi). The adjuvant treatment regimen consisted of weekly paclitaxel at 80 mg/m² and trastuzumab at a loading dose of 4 mg/kg, followed by 2 mg/kg weekly for 12 weeks, followed by trastuzumab weekly at 2 mg/kg or once every 3 weeks at 6 mg/kg to complete a full year of treatment. Patients who underwent lumpectomy received radiation therapy after completing the 12-week chemotherapy, while those with hormone-receptor-positive HER2-positive (“triple-positive”) tumors subsequently received endocrine therapy [59]. Side effects (e.g., cardiac toxicity) were minimal and notably lower compared to anthracycline-containing regimens in other studies. However, a head-to-head comparison of the de-escalated treatment from the APT study and other regimens is not available. In 2023, the 10-year follow-up analysis indicated an iDFS rate of 91.3% (95% CI 88.3–94.4). In patients with HR-positive disease, iDFS rate was 91.6% (95% CI 88.0; 95.4) and in HR-negative tumors, iDFS rate was 90.6% (95% CI 85.1; 96.4). Ten-year recurrence-free survival (RFS) was 96.3% (95% CI 94.3; 98.3; in HR-positive tumors, RFS was 96.2% [95% CI 93.8; 98.7], and in HR-negative disease, RFS was 96.4% [95% CI 93.0; 99.9]). Ten-year OS rate was 94.3% (95% CI 91.8; 96.8), and 10-year BC-specific survival rate was 98.8% (95% CI 97.6; 100) [60]. The longer follow-up results showing a favorable prognosis indicate that adjuvant paclitaxel and trastuzumab represent a reasonable treatment standard, and this has been widely adopted as the recommended therapeutic approach for patients with node-negative HER2-positive eBC and incorporated into national and international guidelines, such as the NCCN [61], ESMO [62], St. Gallen International Consensus [63], and AGO Breast Committee (LoE 2b/B/AGO+) [64]. Since only a small percentage of patients (8.9%) had a tumor size between 2 and 3 cm and/or N1mi disease (1.5%), some guidelines considered the APT regimen as only being suitable for node-negative patients with a tumor size not exceeding 2 cm (Figure 1).

However, the single-arm study design should be subject to critical discussion. In principle, randomized controlled trials are considered the gold standard to assess long-term outcomes or treatment effects. Due to the favorable prognosis of patients with low-risk HER2-positive BC, conducting a randomized trial in this setting may be challenging. Conducting de-escalation studies becomes challenging in the absence of industrial support, primarily due to the low frequency of events. This results in a need for large sample sizes, leading to high costs and rendering the studies difficult to execute.

On the other hand, adjuvant clinical trials involving much higher patient numbers such as TAILORx [69], RxPONDER [70], monarchE [71], NATALEE [72], and OlympiA [73] have been successfully conducted in the last decade. It is important to consider and discuss further limitations: approx. 20% of the recruited patients had a tumor size < 0.5 cm, making them eligible to forego all systemic therapy [29]. Additionally, two-thirds of the recruited patients had HR-positive disease, which is already associated with a favorable prognosis, and received endocrine therapy as part of their systemic therapy. Indeed, the iDFS rate in patients with HR-negative tumors was lower compared to HR-positive tumors, with a total of 10 iDFS events observed in the HR-negative cohort (vs. 19 events in the HR-positive group)

Without doubt, the most critical issue regarding the APT trial is its adjuvant design. Due to the necessity of primary surgical therapy when attempting to adequately assess the nodal status and tumor size, patients are not eligible for neoadjuvant therapy, and it remains unknown whether they might have benefited from a post-neoadjuvant strategy in cases of non-pCR. On the other hand, those diagnosed with either sentinel lymph node metastasis (cN0/pN1) or a tumor larger than 2 cm (cT1/pT2) are usually recommended polychemotherapy with one or two antibodies. Therefore, an optimal pre-therapeutic diagnostic workup including a high-quality ultrasound of the axilla seems crucial to identify patients who are at risk of pathological upstaging. In this context, a potential surgical de-escalation, e.g., omitting sentinel node biopsy in low-risk patients, requires a critical multidisciplinary discussion [74]. Otherwise, a metastatic sentinel node in a patient deemed cN0 who is, in fact, pN+ could remain undiagnosed, with the patient proceeding to receive de-escalated systemic therapy (APT regimen). This simultaneous de-escalation of both surgical and systemic therapy might potentially lead to reduced prognosis. Further, if the initially low-risk HER2-positive tumor becomes high-risk postoperatively, the treatment concept of post-neoadjuvant therapy becomes unfeasible. In patients with HER2-positive early-stage BC who retain residual invasive disease following standard neoadjuvant treatment, the prognosis is notably less favorable compared to those who achieved a pCR, and those receiving post-neoadjuvant T-DM1 in this setting show significantly improved iDFS and OS [30]. 

One of the ongoing trials on the de-escalation of therapy is DECRESCENDO [75]. In this single-arm phase II study, 1065 patients with HR-negative HER2-positive eBC will receive neoadjuvant taxane with trastuzumab plus pertuzumab for 12 weeks. After surgery, the patients who achieved pCR will receive 14 additional cycles of dual HER2 blockade, and patients with non-pCR will be treated with T-DM1 ± anthracycline-based post-neoadjuvant chemotherapy. Another study with a similar design is the CompassHER2 phase II trial conducted by the ECOG-ACRIN Cancer Research Group (NCT04266249).

While the APT, DECRESCENDO, and CompassHER2 studies explore a de-escalation of chemotherapy, other trials focused on the duration of HER2-targeted treatment. The ShortHER phase III trial was designed to assess whether a shorter trastuzumab course is non-inferior to a conventional one-year course [76]. A total of 1254 patients were randomized to a 9-week weekly administration of adjuvant trastuzumab in combination with three cycles of docetaxel, followed by three cycles of FEC chemotherapy vs. four cycles of doxorubicin and cyclophosphamide, followed by four cycles of a docetaxel, along with trastuzumab, continued for a total duration of one year. A total of 672 (54%) patients were node-negative, 383 (30%) had 1–3 positive nodes, and 198 (16%) had 4 or more metastatic nodes. The recent follow-up analysis reported a 10-year DFS of 77% in the long arm and 78% in the short arm (HR 1.06; 90%CI 0.86–1.31) and 10-year OS of 89% in the long and 88% in the short arm (HR 1.15; 90% CI 0.85–1.56) [76]. Despite these comparable long-term outcomes, ShortHER failed to demonstrate non-inferiority of the shorter treatment duration. Therefore, a one-year course of trastuzumab remains the standard treatment. However, it is worth noting that survival differences in node-negative patients (10-year DFS: 81% in the long arm vs. 85% in the short arm, HR 0.74, 90% CI 0.54; 1.04; 10y OS long arm 89%; short arm 95% HR 0.57 90% CI 0.33; 0.99) with 1–3 positive nodes (10-year DFS: 77% in the long arm vs. 79% in the short arm, HR 1.11, 90% CI 0.76; 1.64; 10y OS long arm 92%; short arm 89% HR 1.37 90% CI 0.77; 2.44) were minimal, while patients with four or more metastatic nodes clearly benefitted from a one-year trastuzumab treatment (10y DFS long arm: 63%; short arm: 53% HR 1.84 90% CI 1.24; 2.75; 10y OS long arm: 84%; short arm 64% HR 1.87 90% CI 1.11; 3.14) [76].

Several trials explored a 6-month duration of trastuzumab treatment. At the ESMO Congress 2021, a meta-analysis was presented that included data from five randomized non-inferiority studies [77]. PHARE [78], HORG [79], and PERSEPHONE [80] examined the outcomes of 6 vs. 12 months of adjuvant trastuzumab (*n =* 7950), while SOLD and ShortHER compared 9 weeks with 12 months (*n =* 3428). In the trials comparing 12 vs. 6 months, the 5-year iDFS was 89.3% (12 months) and 88.6% (6 months; HR 1.07 90% CI 0.98–1.17; *p* = 0.002). The non-inferiority limit (HR 1.2) was formally met, leading the authors to conclude the non-inferiority of a 6-month trastuzumab regimen [77]. 

While these studies did not change our treatment standard, they showed that patients are likely to derive the most benefit from trastuzumab in the first weeks/months of therapy. Therefore, when low- or intermediate-risk patients need to discontinue treatment due to toxicity, the results of these trials may be used to provide reassurance. More importantly, they may open the door to a more affordable treatment option for the many patients worldwide who cannot afford the high cost of a full year of trastuzumab.

## 5. Chemo-Free Therapy: Ready for Prime Time? 

In the sense of de-escalation, omitting non-targeted cytotoxic chemotherapy altogether is often hailed as the ultimate goal of oncological research. In this context, chemotherapy-free regimens have recently been evaluated in clinical trials such as WSG ADAPT, PherGAIN, ATEMT, or WSG ADAPT HER2-IV (Table 2). The introduction of immunotherapy, ADCs, and tyrosine kinase inhibitors have considerably broadened the spectrum of potential therapeutic strategies. 

While different clinical settings are currently under investigation, the majority of trials focused on de-escalated neoadjuvant treatment and used pCR as a surrogate endpoint. Some of these studies were conducted using the ADAPT umbrella trial designed by the German WSG Group. The non-inferiority study WSG-ADAPT-HER2+/HR- investigated the efficacy of chemo-free neoadjuvant regimen trastuzumab/pertuzumab compared to paclitaxel plus trastuzumab/pertuzumab in patients with HR-negative HER2-positive eBC [81]. The duration of treatment was 12 weeks in both arms. A total of 36% of patients in the chemotherapy-free cohort achieved a pCR, compared to 91% in the paclitaxel plus trastuzumab/pertuzumab group. After a median follow-up of 60 months, there were numerical but not significant differences between the treatment groups regarding survival endpoints (5-year iDFS: 98% [95% CI 84–100] in the chemotherapy group vs. 87% [95% CI 78–93] in the chemo-free group, HR 0.32, 95% CI 0.07–1.49; *p* = 0.15; relapse-free survival: 98% [95% CI 84–100] vs. 89% [95% CI 79–94], HR 0.41, 95% CI 0.09–1.91; *p* = 0.25); distant DFS: 98% [95% CI 84–100] vs. 92% [95% CI 83–96], HR 0.35, 95% CI 0.04–3.12; *p* = 0.36], OS: 98% [95% CI 84–100] vs. 94% [95% CI 86–97], HR 0.41, 95% CI 0.05–3.63; *p* = 0.43) [81]. 

Interestingly, only two iDFS events occurred in patients achieving pCR (one in each arm). While the confidence intervals in this study are very wide due to the small sample size, and non-inferiority was not formally met, specifically in this study, it is evident that the omission of further chemotherapy did not affect iDFS in patients with pCR after a de-escalated, chemofree, neoadjuvant regimen. Since clinical outcomes were better after chemotherapy-containing treatment, identifying patients most likely to achieve pCR after chemo-free therapy remains challenging. Parameters that might be helpful in this context are among others the molecular subtype and immunohistochemical HER2 expression [81]. Indeed, Graeser et al. were able to show that distinct gene signatures were associated with pCR versus iDFS and that patients with upregulated immune response signatures could be suitable candidates for de-escalation concepts in HR-negative HER2-positive eBC [87].

The combination of neoadjuvant HER2-targeted therapy with endocrine treatment in patients with HR-positive HER2-positive tumors has been explored in the WSG-TP-II study [84]. In this trial, 207 patients were randomized to 12 weeks of dual HER2 blockade (trastuzumab/pertuzumab) in combination with either paclitaxel or endocrine therapy. pCR rate was significantly lower in the chemo-free group (23.7% vs. 56.4%). Interestingly, HER2 messenger RNA levels were associated with tumor response and in patients with the highest quartile of HER2 messenger RNA, pCR rates were comparable in both arms, suggesting that this assay may help to identify patients more likely to respond well to combined endocrine and anti-HER2 therapy.

Another approach to optimized patient selection was tested in the PHERGain trial. Here, imaging tools were used to identify patients who are likely to benefit from de-escalated neoadjuvant treatment [85,86]. In this study, 356 patients with HER2-positive tumors were randomly allocated to two cycles of conventional TCHP regimen (docetaxel/carboplatin/trastuzumab/pertuzumab) vs. chemo-free trastuzumab/pertuzumab, combined with endocrine treatment in triple-positive patients. Early metabolic response was evaluated by [¹⁸F]FDG-PET at baseline and after two cycles. Patients in the standard arm continued to receive TCHP for a further four cycles. In the experimental arm, responders received a further six cycles of chemo-free treatment while non-responders (approx. 20% of patients) were switched to six courses of TCHP. A total of 38% of early responders achieved pCR after eight courses of chemo-free neoadjuvant therapy and these patients had excellent clinical outcomes with 3-year iDFS of 98.8%. However, when all patients in the experimental arm were considered, the iDFS was lower than in the standard arm (95.4% vs. 98.3%), despite the fact that all patients with non-pCR after chemo-free therapy were recommended six courses of TCHP after surgery. The study is ongoing and OS data are pending, but nevertheless PET imaging seems to be a promising tool to distinguish responders from non-responders [85,86].

The potential limitations of chemotherapy-free regimes must be considered, especially in the setting of advanced disease stages. The KRISTINE study randomized patients with stage II-III HER2-positive tumors to a combination of T-DM1 plus pertuzumab vs. docetaxel, carboplatin, and trastuzumab plus pertuzumab [88,89]. Patients allocated to T-DM1 plus pertuzumab continued the same treatment after surgery, and patients who received TCHP received adjuvant trastuzumab/pertuzumab. pCR rates were lower in the experimental arm (44.4%) compared to TCHP (55.7%, *p* = 0.016). After a median follow-up of 37 months, the risk of an EFS event was higher with T-DM1 plus pertuzumab (HR 2.61, 95% CI 1.36–4.98) and more locoregional progressions were observed before surgery (6.7% vs. 0%). iDFS rates after surgery were similar between arms (HR 1.11, 95% CI 0.52–2.40). While grade ≥ 3 adverse events were less common with T-DM1 plus pertuzumab (31.8% vs. 67.7%), toxicity leading to treatment discontinuation after surgery occurred more frequently in the T-DM1 plus the pertuzumab arm (18.4% vs. 3.8%).

Current ongoing studies investigate further questions such as the comparison of neoadjuvant T-DXd vs. chemotherapy plus dual HER2 blockade (trastuzumab/pertuzumab). The ADAPT-HER2-IV trial randomized patients with low- to intermediate-risk HER2-positive tumors to 12 weeks of neoadjuvant T-DXd vs. paclitaxel plus trastuzumab/pertuzumab and those with intermediate- to high-risk tumors to 18 weeks of T-DXd vs. conventional neoadjuvant chemotherapy with dual HER2 blockade. The trial is designed to serve as a superiority trial to demonstrate the higher pCR rates observed in both clinically relevant subgroups treated by T-DXd. Recruitment began in 2023. 

Even though the concept of chemotherapy-free regimens seems likely to become an option, at least for some carefully selected patients, survival data from most trials are still pending. ADCs appear to be especially promising candidates for a de-escalated treatment due to their favorable safety and enhanced efficacy [90]. However, optimal duration, dosage, and combination partners for an ADC-based neoadjuvant therapy remain to be cleared.

## 6. Conclusions

In recent decades, an increasing number of targeted oncological therapeutics has been approved for use based on molecular parameters such as receptor expression, amplification, and mutation status. Due to the introduction of numerous targeted anti-HER2 strategies, HER2-positive breast carcinoma, previously considered to be an entity with a particularly poor prognosis, became an easily treatable disease and one of the subtypes with the best clinical outcomes. This groundbreaking medical development began with the approval of trastuzumab following the pivotal study by Slamon et al. in the year 2000 and marked the beginning of a new era in targeted tumor therapy. The gold standard for HER2-positive BC with residual invasive disease following neoadjuvant therapy is T-DM1 administered in the post-neoadjuvant setting. The most recent survival data, presented at the SABCS 2023, showed that T-DM1 improves OS in these high-risk patients [30].

It remains to be seen whether the data from the TruDy (Destiny Breast 05) study, a head-to-head comparison of T-DM1, and another ADC T-DXd in cases of non-pCR will challenge this standard of care, potentially establishing a role for T-DXd in the post-neoadjuvant setting in the future [49]. Currently, T-DXd is only approved for HER2-positive and HER2-low metastatic BC [22,50]. However, medical history demonstrates that new therapeutic options usually prove their efficacy in advanced stages before being introduced into the (neo-)adjuvant treatment. Early clinical management in dealing with new side effects is considered essential, as potentially life-threatening ADC-mediated complications must be approached with particular caution in the curative setting.

Therapeutic de-escalation is an important and forward-looking approach in oncology, with an increasing significance in the field of both surgical senology and systemic therapy for patients with BC. The right balance between selecting an appropriate systemic therapy and avoiding morbidity to enhance patients’ quality of life is particularly important in the case of low-risk tumors. In this context, the “APT regimen”, i.e., 12 weeks of paclitaxel in combination with a 1-year course of trastuzumab, has been widely adopted in the global guidelines as the standard of care for low-risk patients, even if the main critical issue is the absence of the option for post-neoadjuvant therapy if the initially low-risk HER2-positive tumor becomes a high-risk tumor postoperatively [59,60,61,62,63,64,65]. 

One of the most exciting opportunities created by the introduction of targeted therapy is the potential to limit chemotherapy-related toxicity. Several ongoing studies, including WSG ADAPT umbrella trials [81], PherGAIN [85], ATEMPT [83], and WSG ADAPT HER2-IV (NCT05704829), are currently assessing chemo-free regimens strategies aiming to de-escalate therapy in the field of oncology. Despite the promising early results obtained from these studies, the combination of anti-HER2 treatment with chemotherapy backbone remains the standard of care. 

The evolution of HER2-directed therapy in BC is a success story and achieving a curative treatment, even for HER2 metastatic BC, is no longer unthinkable. While a chemotherapy-free treatment regimen may be considered for certain patients, survival data from most trials are still pending. Currently, the possibilities for ultimate systemic de-escalation in the discussed patient population are quite limited. However, in the future, the expansion of ultimate de-escalation appears to be possible, especially with ADCs emerging as suitable therapeutics due to their biochemical structure, facilitating the transition from non-targeted chemotherapy towards targeted tumor therapy.

## Figures and Tables

**Table 1:** Overview of the most important studies in HER2-positive eBC.

Study and Patient Number (n)	Design	Conclusion
HERA [26]; *n =* 5102	International, multicenter, open-label, phase III, randomized trial. Adjuvant setting. Enrollment between December 2001 and June 2005. Women with HER2-positive eBC. After primary therapy (including, surgery, chemotherapy, and radiotherapy as indicated): patients randomly assigned (1:1:1). Trastuzumab for 1 year. Trastuzumab for 2 years (with the same dose schedule). Observation group.	Trastuzumab for one year improves long-term DFS and OS compared to observation. No additional benefit of two years of trastuzumab. Long-term iDFS (after median follow-up 11 years): Observation: estimates of 10-year disease-free survival 63%. Trastuzumab for 1 year: estimates of 10-year disease-free survival 69%. HR 0.76; *p* = 0.0001; 95% CI 0.66; 0.8. Trastuzumab for 2 years: Estimates of 10-year disease-free survival 69%. HR 0.99 *p* = 0.86 95% CI 0.85; 1.14. OS (after median follow up 11 years): observation group 73% Trastumab for 1 year: 79%. HR 0.76; *p*-value 0.0005; 95% CI 0.65; 0.88. Trastuzumab for 2 years: 80%. HR 1.05; *p* = 0.63; 95% CI 0.86; 1.28.
APHINITY [27]; *n =* 4805	International, randomized, placebo-controlled, phase III, double-blind, multicenter trial. Adjuvant setting. Enrollment between November 2011 and August 2013. Patients with HER2-positive node-positive or high-risk node-negative BC: randomly assigned (1:1) to 1-year pertuzumab or placebo added to standard adjuvant chemotherapy and 1-year trastuzumab.	Trastuzumab and pertuzumab in combination with chemotherapy improves iDFS compared to trastuzumab plus chemotherapy. iDFS (after median follow-up 6 years): HR 0.76 (95% CI, 0.64–0.91). Pertuzumab 91% iDFS; 88% iDFS for placebo groups. OS: no statistical significance 95% (pertuzumab vs. 94% placebo).
NeoSphere [28]; *n =* 417	International, multicenter, open-label, phase II randomized trial. Neoadjuvant setting. Treatment-naive adults with locally advanced, inflammatory, or early-stage HER2-positive BC randomly assigned (1:1:1:1) to 4 neoadjuvant cycles of: Trastuzumab plus docetaxel (Group A). Pertuzumab and trastuzumab plus docetaxel (Group B). Pertuzumab and trastuzumab (Group C). Pertuzumab and docetaxel (Group D).	Combination of pertuzumab, trastuzumab and docetaxel is associated with significantly higher pCR compared to other arms. PFS and DFS at 5-year follow-up seem beneficial if neoadjuvant pertuzumab is combined with trastuzumab and docetaxel. 5-year PFS: Group A: 81% (95% CI 71; 87). Group B: 86% (77; 91). Group C: 73% (64; 81). Group D: 73% (63; 81). HR 0.69 (95% CI 0.34; 1.40 Group B vs. Group A, 1.25 (0.68; 2.30) Group C vs. Group A, 2.05 (1.07; 3.93) Group D vs. Group B. iDFS: Group A: 81% (95% CI 72; 88). Group B: 84% (72; 91). Group C: 80% (70; 86). Group D: 75% (64; 83).
KATHERINE [29,30]; *n =* 1486	International, multicenter, phase III, open-label, randomized trial. Post-neoadjuvant setting. Between April 2013 and December 2015. HER2-positive eBC with residual invasive disease in the breast and/or axilla at surgery after receiving neoadjuvant therapy containing a taxane (with or without anthracycline) and trastuzumab. Adjuvant T-DM1 or trastuzumab for 14 cycles.	50% lower risk of recurrence of invasive BC or death with adjuvant T-DM1 than with trastuzumab alone. Significantly improved OS at 7 years (89.1% in the T-DM1 arm vs. 84.4% in trastuzumab arm, HR 0.66; 95% CI 0.51, 0.87; *p* = 0.0027). Final iDFS benefit at 7 years 80.8% vs. 67.1%, HR 0.54, 95% CI 0.44, 0.66.
ExteNET [31]; *n =* 2840	Randomised, double-blind, placebo-controlled, phase 3 trial. Her2 + stage 1-3c (modified to stage 2-3c in February, 2010) operable breast cancer, completed neoadjuvant and adjuvant chemotherapy plus trastuzumab with no evidence of disease recurrence or metastatic disease. 12 months’ neratinib 240 mg/day p.o. (pan-HER tyrosine kinase inhibitor) vs. placebo after neoadjuvant and adjuvant treatment with trastuzumab for one year i.v.	1 year of neratinib significantly improves 5-year invasive-disease-free survival and an absolute 8-year OS benefit after trastuzumab-based adjuvant therapy in women with HER2-positive breast cancer. iDFS benefits at 5 years 5.1% in HR+/initiated treatment post-trastuzumab ≤ 1-year (HR, 0.58; 95% CI, 0.41; 0.82). Absolute OS benefit at 8 years: 2.1%; HR 0.79; 95% CI, 0.55; 1.13.

**Table 2:** Overview of the most important clinical trials for chemotherapy-free regimens.

Study and Patient Number	Design	Conclusion
WSG-ADAPT-HER2+/HR– [81,82]; *n =* 134	Investigator-initiated, multicenter, open-label, randomized, non-inferiority phase II trial as part of the ADAPT umbrella trials. Neoadjuvant setting. HR-negative HER2-positive eBC. 12 weeks of trastuzumab/pertuzumab vs. trastuzumab/pertuzumab plus paclitaxel.	pCR rate higher in paclitaxel arm (90.5% vs. 36.3%). Post-neoadjuvant chemotherapy did not affect iDFS in patients with pCR. Low-risk early stages might not need chemotherapy if pCR is gained by dual HER2 blockade.
ATEMPT [83]; *n =* 497	Multicenter, randomized phase II trial. Adjuvant setting. Stage I HER2-positive eBC. 1 year T-DM1 vs. 12 weeks paclitaxel plus 1 year trastuzumab.	No difference in 3-year iDFS. Less neuropathy (11% vs. 23%, *p* = 0.03) and alopecia (0% vs. 41%, *p* < 0.001) in TDM-1 group but no difference in CRTs (46% vs. 47%).
WSG TP II [84]; *n =* 207	Multicenter, randomized, open-label, non-comparative, phase II trial. Neoadjuvant setting. HR-positive HER2-positive eBC. 12 weeks of paclitaxel/trastuzumab/pertuzumab vs. endocrine therapy/trastuzumab/pertuzumab.	Higher pCR rate in paclitaxel arm (57% vs. 24%, OR 0.24, 95% CI: 0–0.46, *p* < 0.001). Survival data pending.
PHERGain [85,86]; *n =* 356	Multicenter, randomized, open-label, non-comparative, phase II trial. Neoadjuvant setting. HER2-positive eBC. TCHP (Group A) vs. trastuzumab/pertuzumab (+ endocrine therapy in HR-positive tumors) (Group B); ¹⁸F-FDG-PET to assess response after 2 cycles and identify patients most likely to benefit from chemotherapy-free treatment.	3-year iDFS Group B: 95.4% (95% CI, 92.8; 98.0); pCR rate of PET responders 37.9% (*p* < 0.001, based on a null hypothesis of ≤20% rate); 20.4% non-responders.
WSG ADAPT HER2-IV (NCT05704829); *n =* 402	Ongoing multicenter, interventional, prospective, two-arm, randomized, open-label phase II trial. Neoadjuvant setting. HER2-positive eBC. Low- to intermediate-risk: (e.g., node-negative patients with cT1-2 tumors): 12 weeks of T-DXd vs. paclitaxel + trastuzumab + pertuzumab. Intermediate- to high-risk: 18 weeks of T-DXd vs. TCHP.	Start 12/2023, estimated completion date 06/2028.
